# Aptamers: Functional-Structural Studies and Biomedical Applications

**DOI:** 10.3390/ijms23094796

**Published:** 2022-04-27

**Authors:** Romualdo Troisi, Filomena Sica

**Affiliations:** Department of Chemical Sciences, University of Naples Federico II, Complesso Universitario di Monte Sant’Angelo, Via Cintia, I-80126 Naples, Italy; romualdo.troisi@unina.it

Aptamers are synthetic molecules of different natures (mostly, DNA or RNA) that recognize a target molecule with high affinity and specificity. Based on their peculiar properties, aptamers have the great potential to be the ideal solution to several problems covering a wide array of fields. Indeed, since the development of the systematic evolution of ligands by exponential enrichment (SELEX) procedure in the 1990s, several aptamers have been selected as biomedical agents or for the development of analytical methods. However, multiple challenges such as the optimization of selectivity, stability, delivery, and long-term safety, as well as reproducibility, still need to be addressed for a full exploitation of the enormous potential of aptamers. The purpose of this Special Issue is to provide an overview of all recent rapidly growing developments of the research in this field.

Despite promising results, a bottleneck is still the selection of high-quality aptamers against relevant targets. Recently, technological progress was implemented in the SELEX process to enhance the performance of aptamer discovery. In particular, HiTS-FLIP is a technique allowing several million DNA sequences to be analyzed in parallel and without bias thanks to the coupling of high-throughput sequencing with fluorescence-based affinity and specificity assays [1]. Likewise, the combined use of the Cell-SELEX technology and fluorescently labeled aptamers represents an advanced tool for the identification of molecular targets in their native state on whole live cells [2]. A great contribution can also be provided by the artificial intelligence methods that, by predicting the binding ability of aptamers, could help to rapidly identify the potential candidates from a vast number of sequences [3]. 

Understanding the mechanisms that govern the specificity and the high-affinity typical of the aptamer–target interactions is fundamental to improve the aptamer properties. In this context, a comprehensive analysis of the structure–activity relationship, which can rely on many structural techniques, is of a great value [4]. In particular, several studies allowed to identify the relationship between specific structural motifs and the stability and/or biological activity of the aptamers [5]. Moreover, the structural analyses also enable the comparison of the mode-of-action of the aptamers with respect to other natural or synthetic ligands, as well as the evaluation of peculiar allosteric effects that they can induce on the interacting target molecule [6]. 

The susceptibility to degradation by nucleases, fast renal clearance, low thermal stability, and the limited functional group diversity slow down the realization of promising aptamer applications as therapeutics at the clinical level. Diverse strategies can be applied in aptamer modification in the SELEX process or by post-SELEX procedures to overcome these drawbacks and to increase the binding affinity [4,7,8,9] or the selective delivery to specific cellular targets [10].

As concerns the roles of aptamers, numerous and varied are their applications in the therapeutic [6,7,8,9,11,12] and diagnostic [2,9,13] fields. For example, various aptamers that modulate the blood coagulation pathway, exhibiting the ability to treat cardiovascular diseases, blood disorders, and cancers, have been selected, studied, and modified [6,7,8,11]. Promising results have also been obtained using a complex formed by a DNA aptamer and vitamin C in the treatment of cognitive impairments associated with the vascular dementia [12]. Furthermore, the selection of aptamers able to recognize different pathogens could inspire aptamer-functionalized tools for the fast diagnosis of infections and/or the fluid filtration [13]. Finally, potential applications of aptamers for personalized gene therapy have recently been proposed including the use of functional RNA aptamers containing both lipid raft-targeting motif and a therapeutic motif to selectively introduce RNAs into exosomes [10]. 

The collection of papers and reviews presented in this Special Issue provides interesting examples of the novel and unique achievements about aptamers that are being discovered day-by-day. 
